# Supplementary material for: Single-cell multiomics decodes regulatory programs for mouse secondary palate development
Source: Nat Commun. 2024 Jan 27;15:821. doi: 10.1038/s41467-024-45199-x (PMC10821874; doi:10.1038/s41467-024-45199-x)
Supplement: Supplementary file 1 — Supplementary Information [file 41467_2024_45199_MOESM1_ESM.pdf]

Supplementary information

Single-cell multiomics decodes regulatory programs for mouse secondary palate development

Fangfang Yan[1], Akiko Suzuki[2,3,†], Chihiro Iwaya[2,3,†], Guangsheng Pei[1], Xian Chen[1], Hiroki Yoshioka[2,3], Meifang Yu[1], Lukas M. Simon[4,*], Junichi Iwata[2,3,*], Zhongming Zhao[1,5,*]

1. Center for Precision Health, School of Biomedical Informatics, The University of Texas Health Science Center at Houston, Houston, TX 77030, USA

2. Department of Diagnostic and Biomedical Sciences, School of Dentistry, The University of Texas Health Science Center at Houston, Houston, TX 77054, USA

3. Center for Craniofacial Research, The University of Texas Health Science Center at Houston, Houston, TX 77054, USA

4. Therapeutic Innovation Center, Baylor College of Medicine, Houston, TX 77030, USA

5. Human Genetics Center, School of Public Health, The University of Texas Health Science Center at Houston, Houston, TX 77030, USA

[†]Authors shared the second authorship

[*]Correspondence: lukas.simon@bcm.edu (L.S.), junichi.iwata@uth.tmc.edu (J.I.), zhongming.zhao@uth.tmc.edu (Z.Z.)

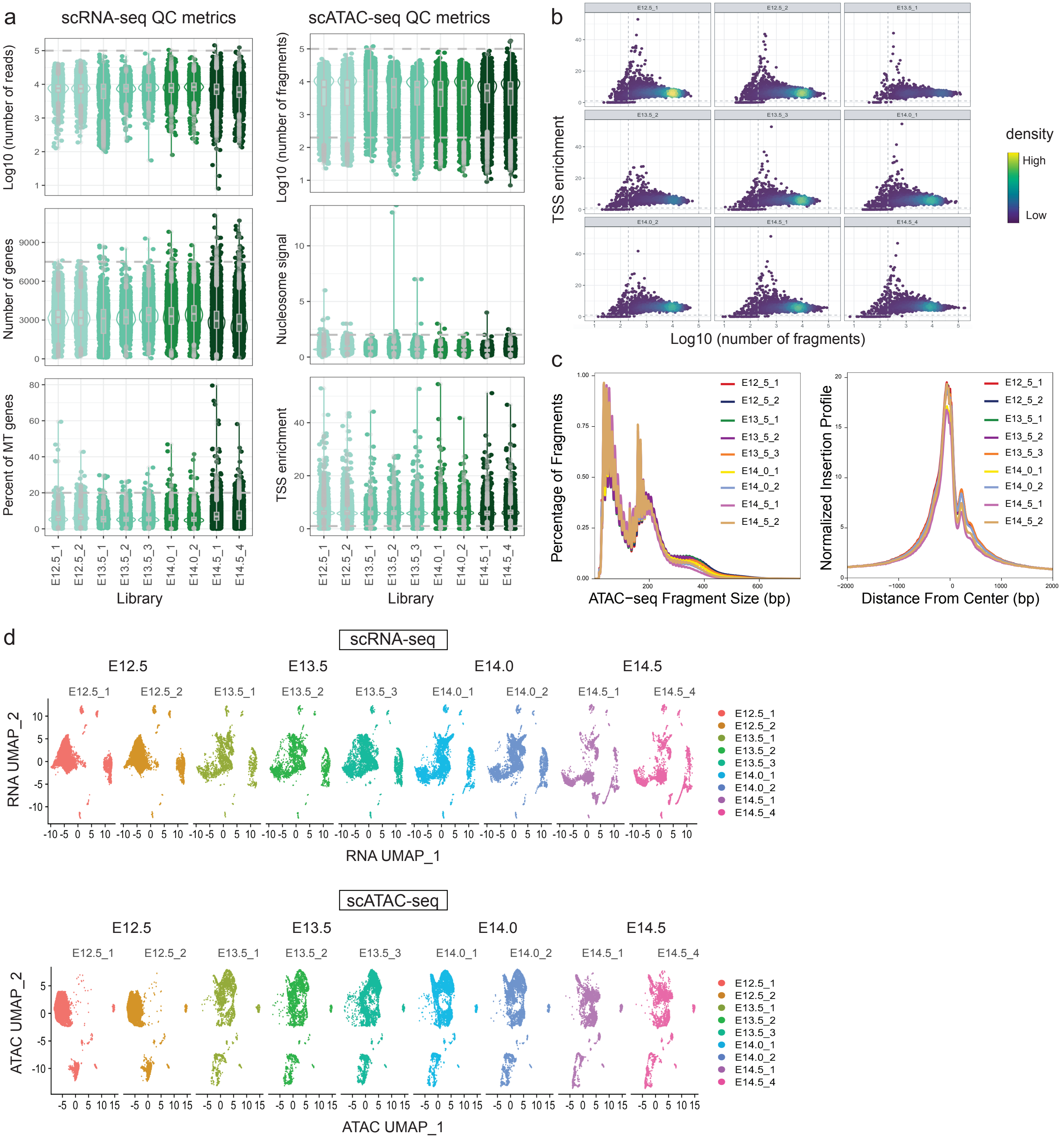

Supplementary Fig. 1. Data quality of snATAC-seq and snRNA-seq libraries. a Violin plots show the distribution of several scRNA-seq (left) and scATAC-seq (right) quality metrics for each cell. scRNA-seq metrics include the total number of RNA reads, total number of genes detected, and percentage of counts from mitochondrial genes (from top to bottom). scATAC-seq metrics include total number of ATAC fragments, nucleosome signal, and transcription start site (TSS) enrichment (from top to bottom). Grey dashed lines represent filtering thresholds. Each plot is separated by developmental stage (x-axis). E: embryonic day. **b** Hexbin plots show density of cells stratified by total number of fragments (x-axis) and TSS enrichment (y-axis). Grey dashed lines represent filtering thresholds. Color represents density. **c** Left: Density plot shows the distribution of fragment size for each sample. Right: Plot shows the normalized fragment count in each positive relative to TSS (bp) for each sample. **d** UMAP visualization based on scRNA-seq (top) and scATAC-seq (bottom) data represent cells colored by each sample and separated by developmental stage.

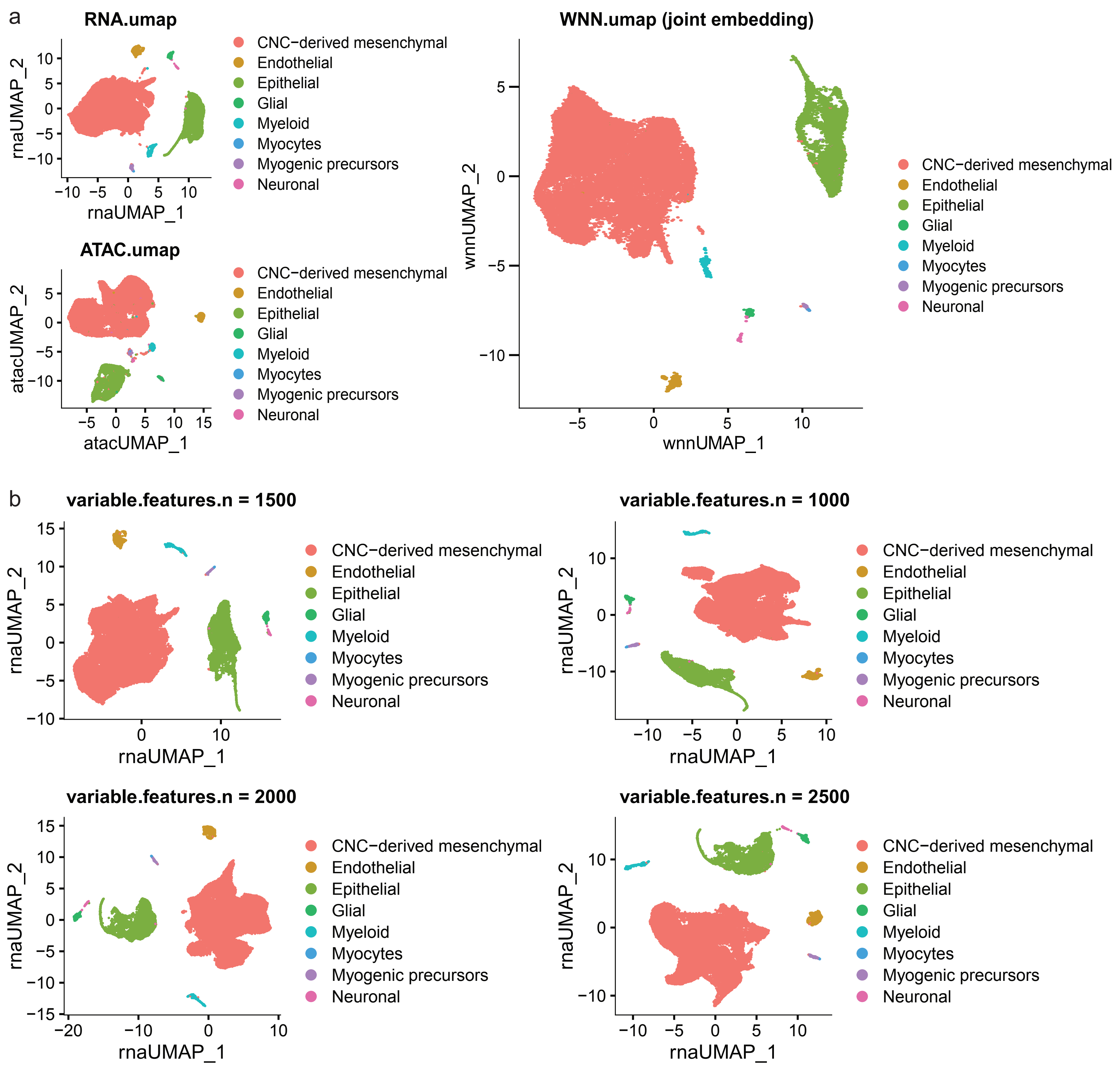

Supplementary Fig. 2. Embedding and cell type annotation is robust across modalities and highly variable gene counts. a UMAP visualization based on different modalitie. **b** UMAP visualization based on different number of highly variable genes. Cell types were annotated independently based on canonical marker expression. The annotation results are robust across various cutoffs for the number of highly variable genes.

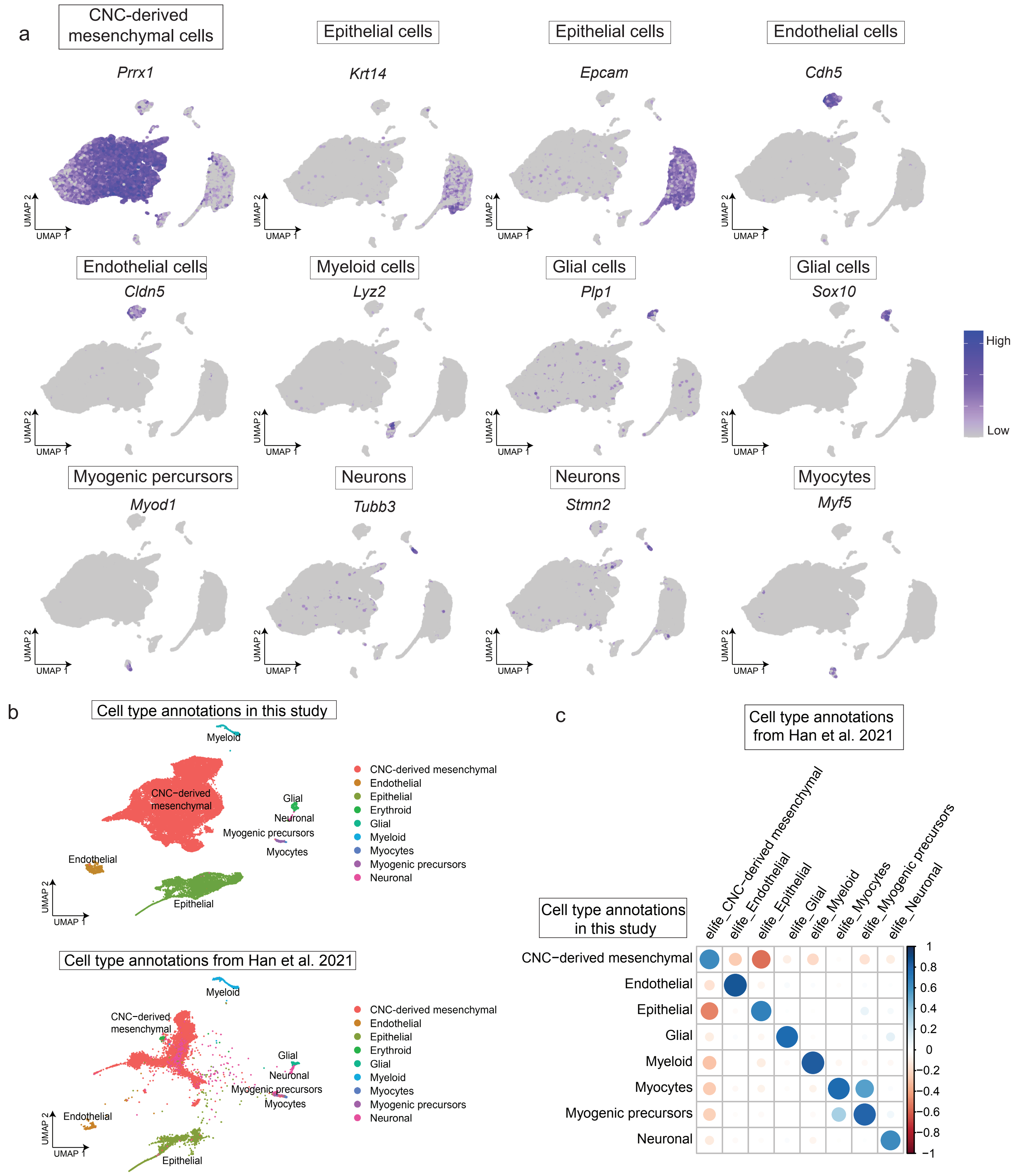

Supplementary Fig. 3. Integration of external snRNA-seq dataset validates cell type annotations. a Feature plots show marker gene expression in each major cell type. **b** External data from Han et al. 20021 was processed and integrated with the snRNA-seq data in this study. Integrated UMAP was separated for visualization purposes and showed correspondence between cell type annotations in this study (top) and Han et al (bottom). **c** Correlogram shows the correlation of cell-type specific fold-changes between cell type annotations in this study (rows) and Han et al. 2021 (columns). Each circle represents the correlation of the gene expression profile between cell types. Positive correlations are displayed in blue and negative correlations in red color. Color intensity and the size of the circle are proportional to the correlation coefficients.

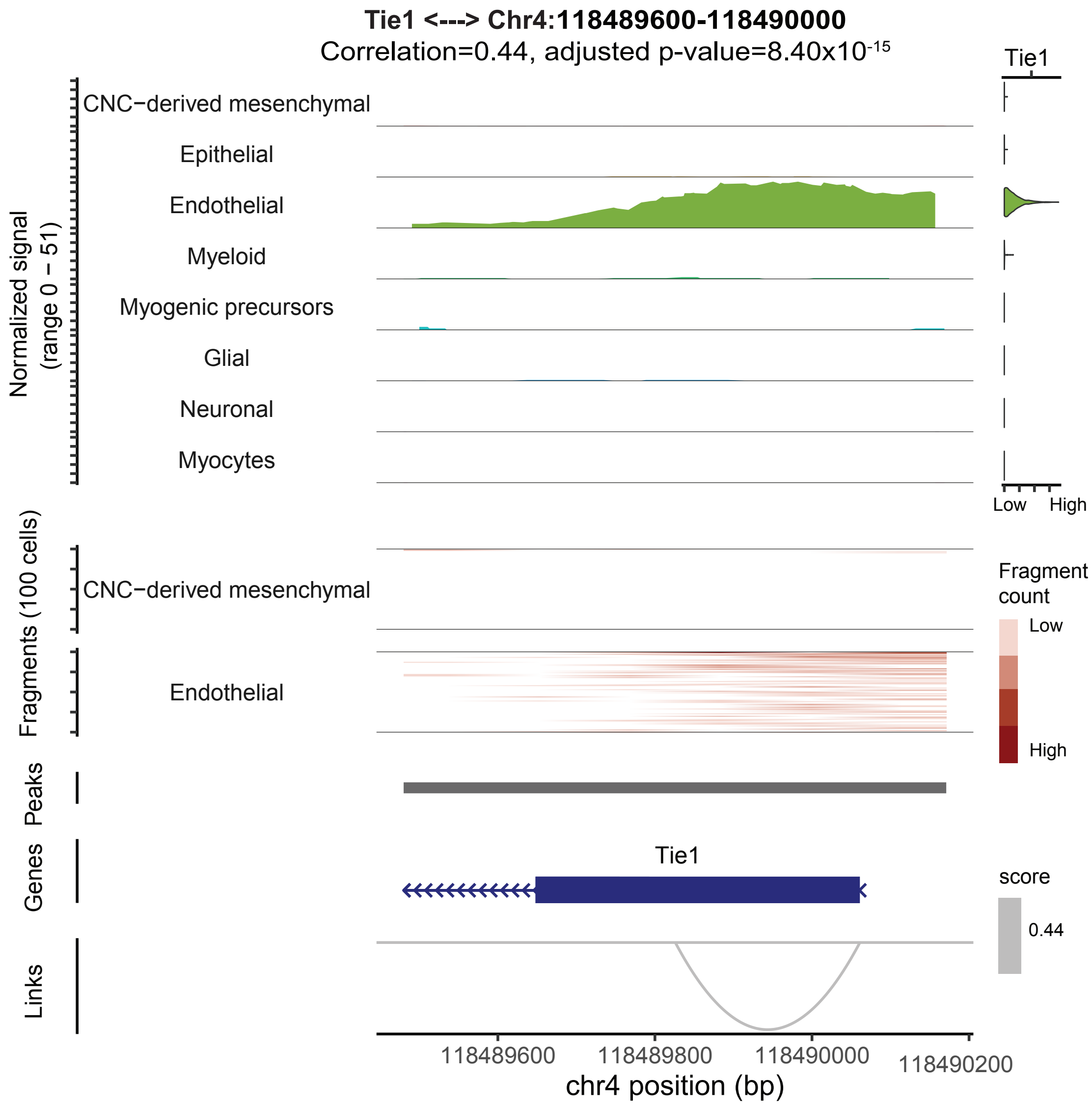

Supplementary Fig. 4. Visualization displaying positively peak-gene linkage specifically in endothelial cells. Left: Genome Browser visualization of aggregated chromatic accessibility at the chr4:118489600-118490000 locus for each major cell type, coupled with Tie1 gene expression. Arcs at the bottom denotes linkage between Tie1 and Chr4:118489480-118490171.

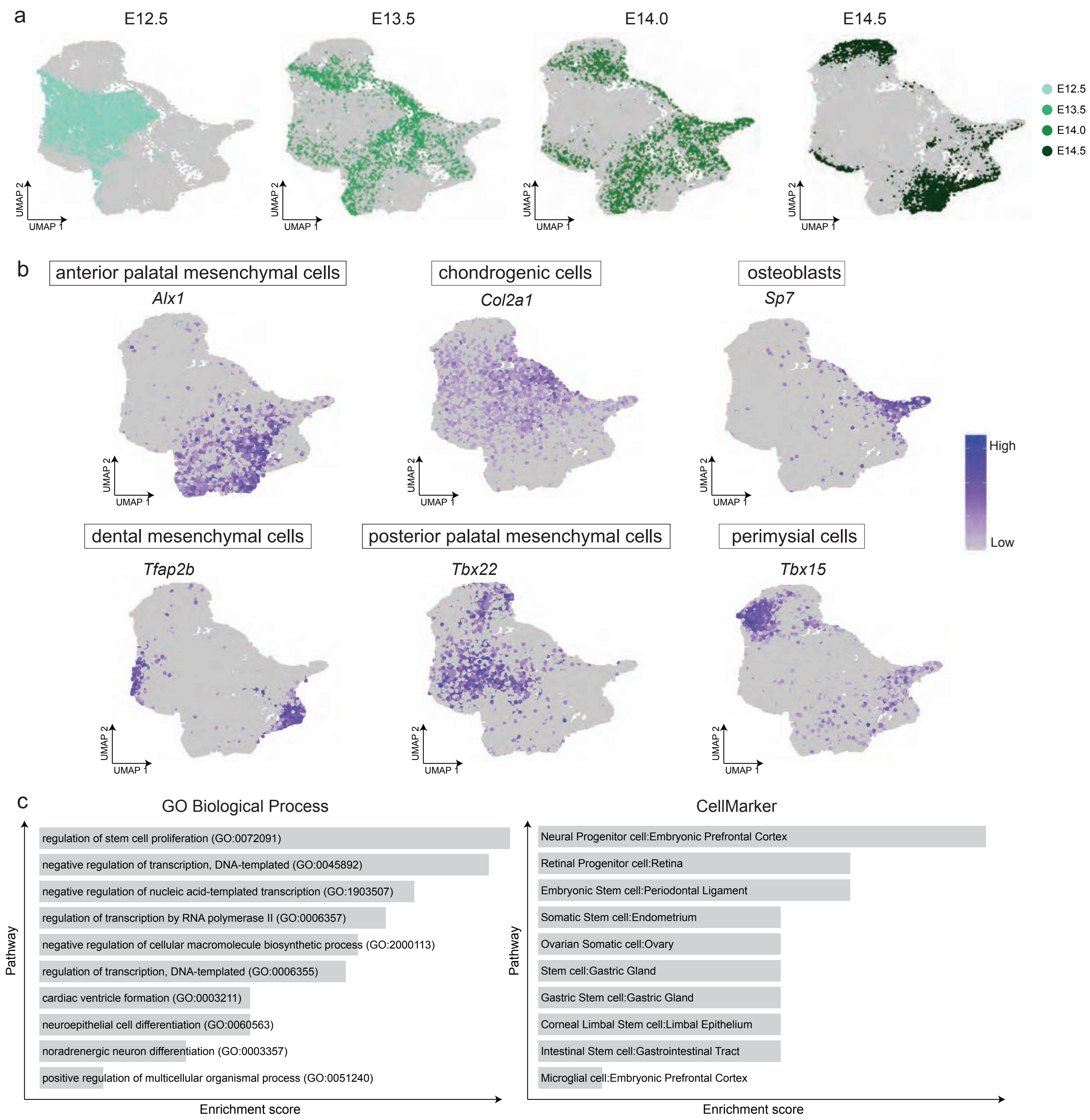

Supplementary Fig. 5. Cell subtype annotations for CNC-derived mesenchymal cells. a UMAP visualization of CNC-derived mesenychmal cells colored by developmental stage. **b** Feature plot of marker genes specific to each subtype of CNC-derived mesenchymal cells. **c** Barplot shows enrichment results for genes from cluster 5 in Figure 3A based on gene ontology (GO) Biological Process pathways (left) and CellMarker cell types (right).

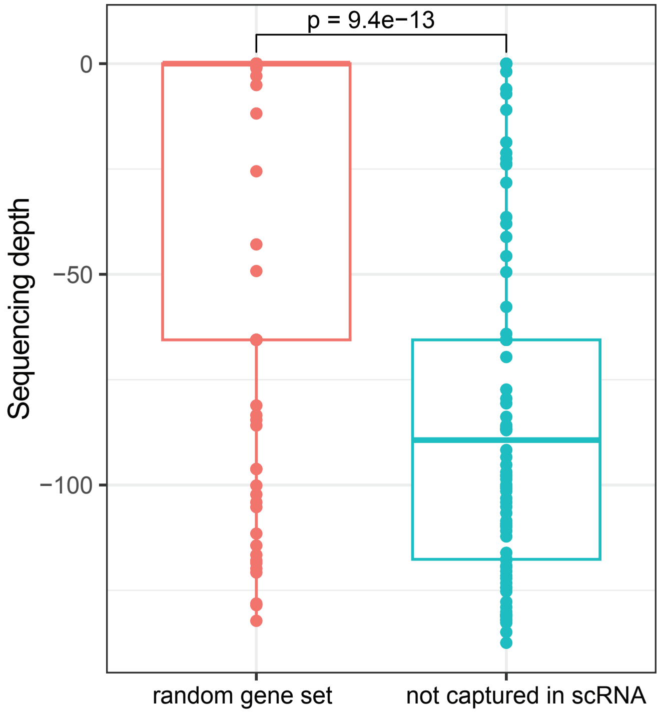

Supplementary Fig. 6. Genes differentially expressed in bulk RNA-seq but undetected in scRNA-seq are lowly expressed. The box plot illustrates sequencing depth (y-axis) across different gene sets (x-axis). The p-value is indicated on the plot.

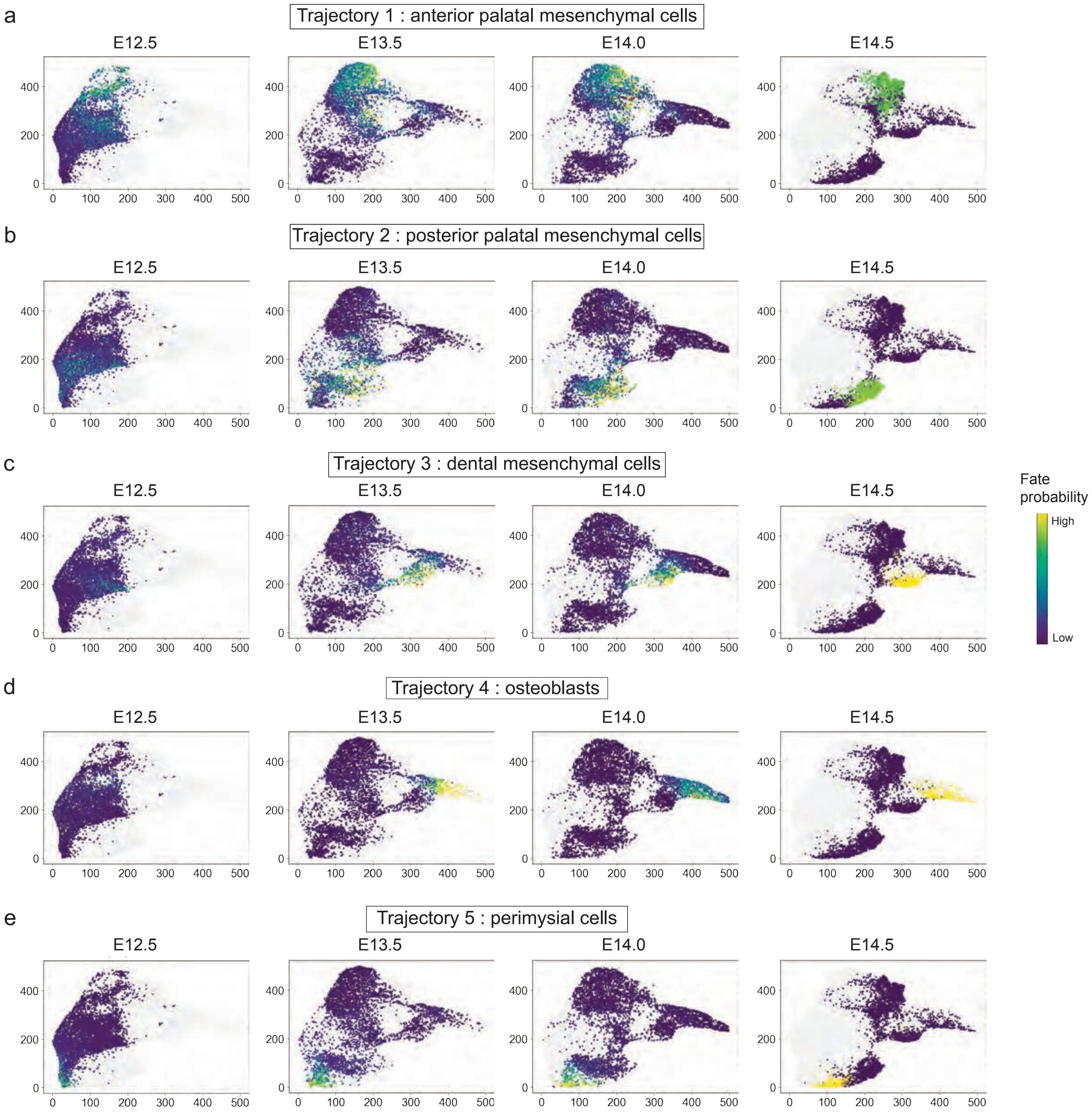

Supplementary Fig. 7. Trajectory analysis reveals continuous differentiation of CNC-derived multipotent cells into distinct terminal states. UMAP visualization of CNC-derived mesenchymal cells separated by time-point and is colored by fate probabilities to each trajectory (high: yellow, low: purple). **a** Trajectory 1: anterior palatal mesenchymal cells, **b** Trajectory 2: posterior palatal mesenchymal cells, **c** Trajectory 3: dental mesenchymal cells, **d** Trajectory 4: osteoblasts, **e** Trajectory 5: perimysial cells.

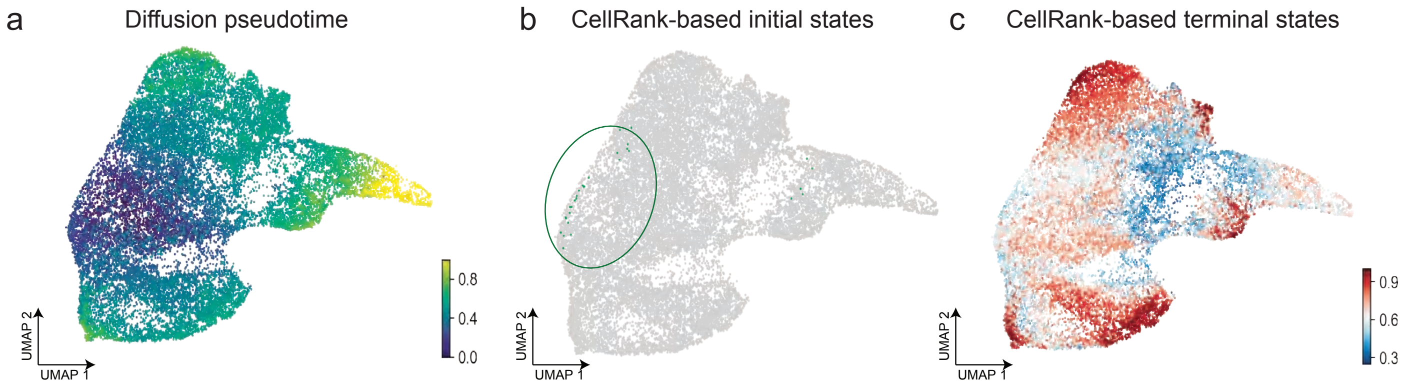

Supplementary Fig. 8. CellRank based on RNA velocity validated the inferred trajectories. UMAP visualization of CNC-derived mesenchymal cells colored by **(A)** diffusion pseudotime, **(B)** initial states, and **(C)** terminal state likelihoods. Red and blue colors represent high and low terminal state likelihood, respectively.

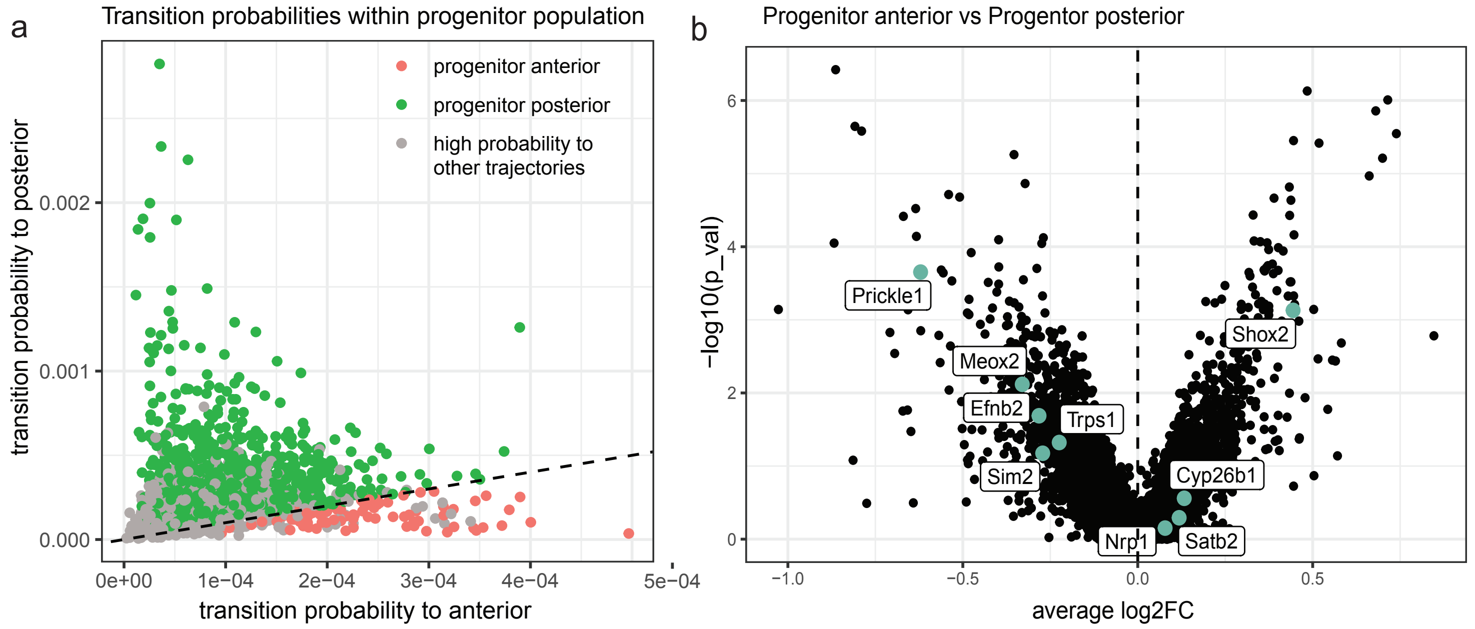

Supplementary Fig. 9. Progenitor cells with high transition probabilities displayed higher anterior-specific genes expressions. a Scatter plot of transition probability to anterior (x-axis) and posterior (y-axis) of progenitor cells. **b** Volcano plot shows average log2 fold change (x-axis) and -log10 adjusted p-value (y-axis) of differentially expressed genes between progentor anterior cells and progenitor posterior cells.

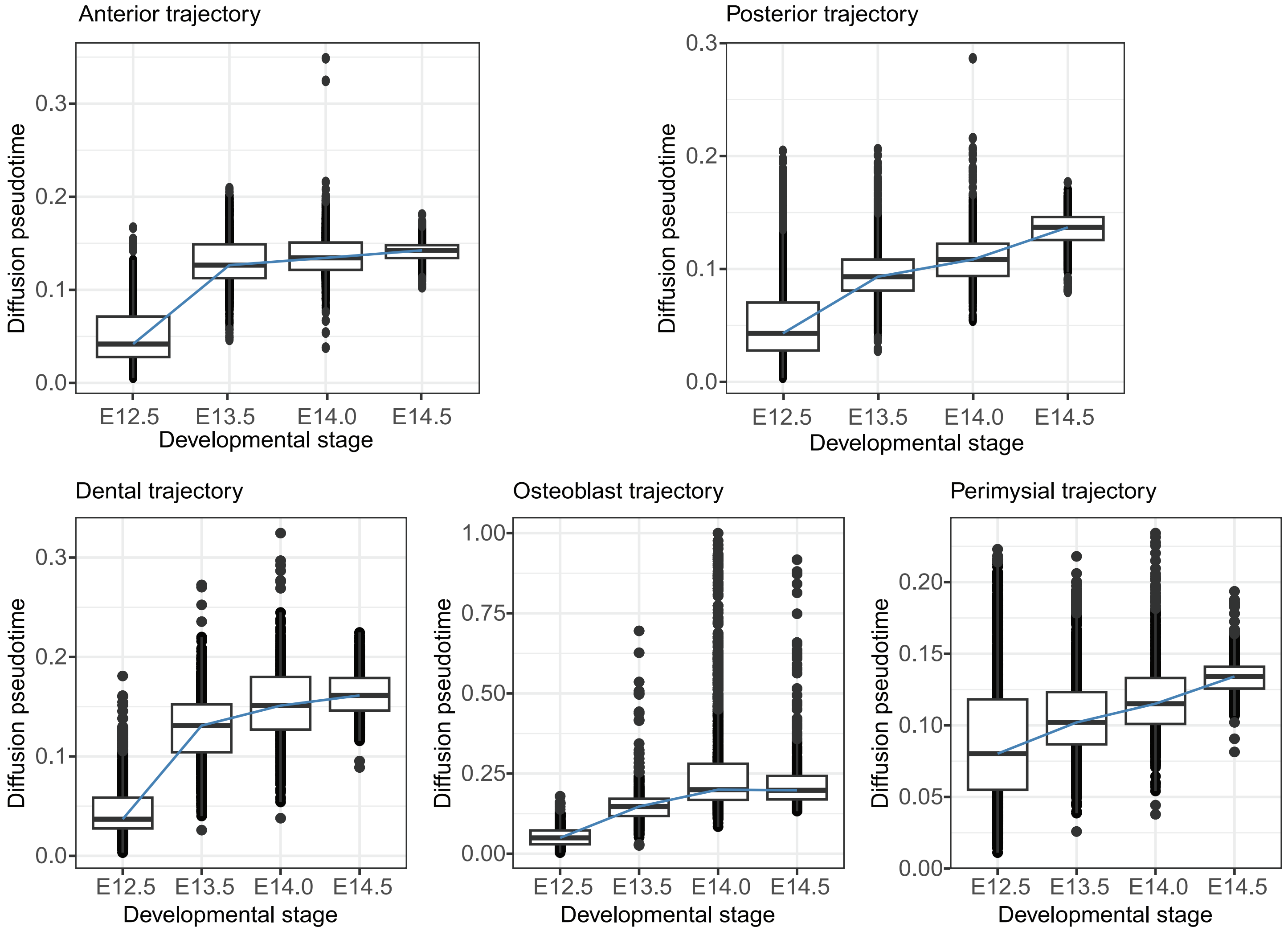

Supplementary Fig. 10. The diffusion pseudotime is consistent with real developmental stage for each trajectory. The boxplot depicts the diffusion pseudotime (y-axis) and real time (x-axis) for each cell in each trajetory.

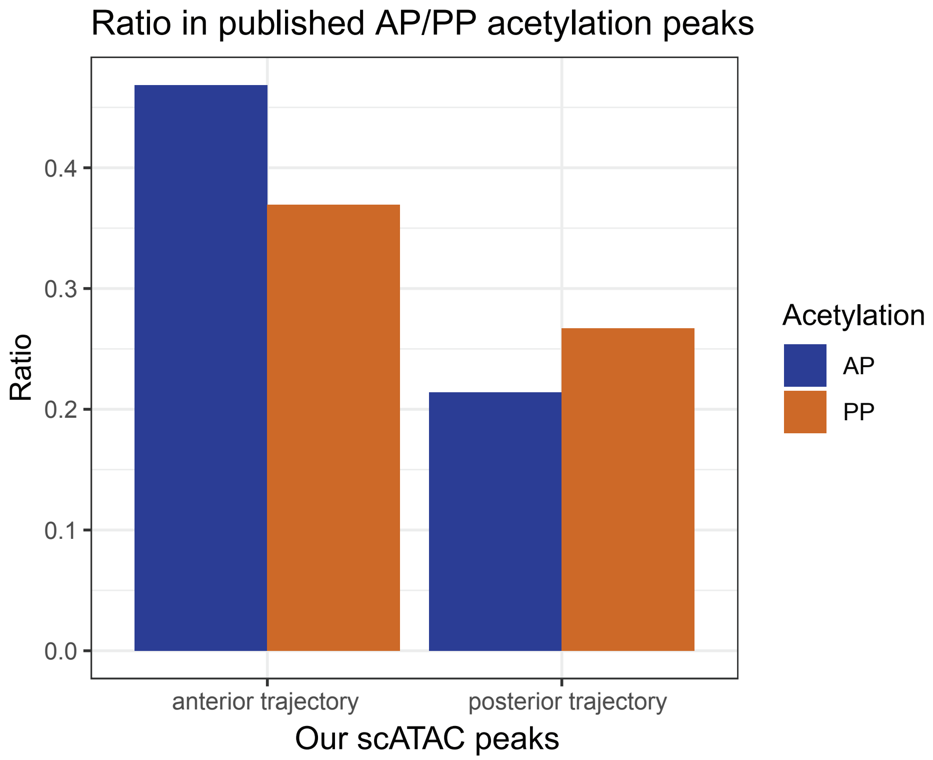

Supplemental Fig. 11. The H3K27 acetylation profiles in the anterior and posterior palate validated the inferred trajectories. Bar plot shows overlap (y-axis) between the scATAC anterior and posterior peaks and the corresponding anterior and posterior acetylation tracks (x-axis).

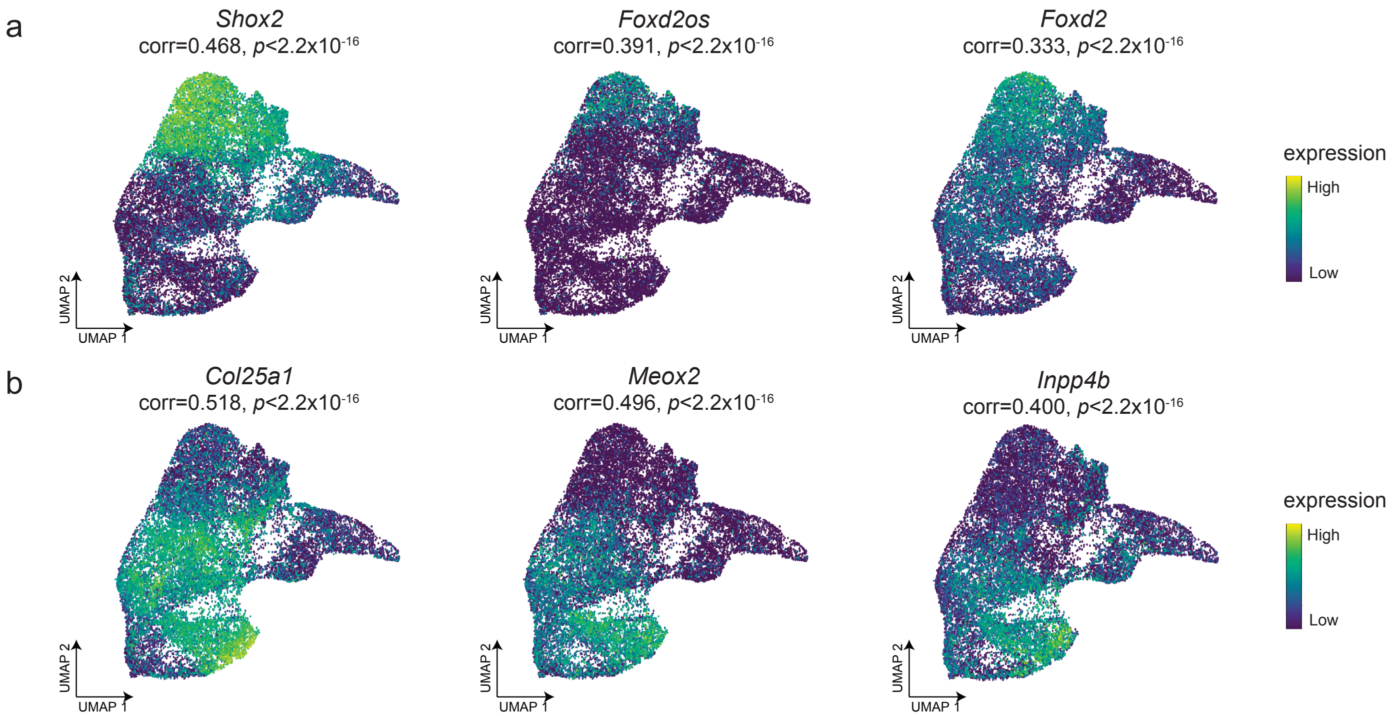

Supplementary Fig. 12. Driver genes were identified for the anterior and posterior trajectory, respectively. Feature plot show expression pattern of top driver genes for **(A)** anterior and **(B)** posterior trajectory. Each dot represent a cell and is colored by gene expression (yellow: high, dark brown: low). Gene name, adjusted p-value, and correlation with fate probabilities was annotated on top of each plot.

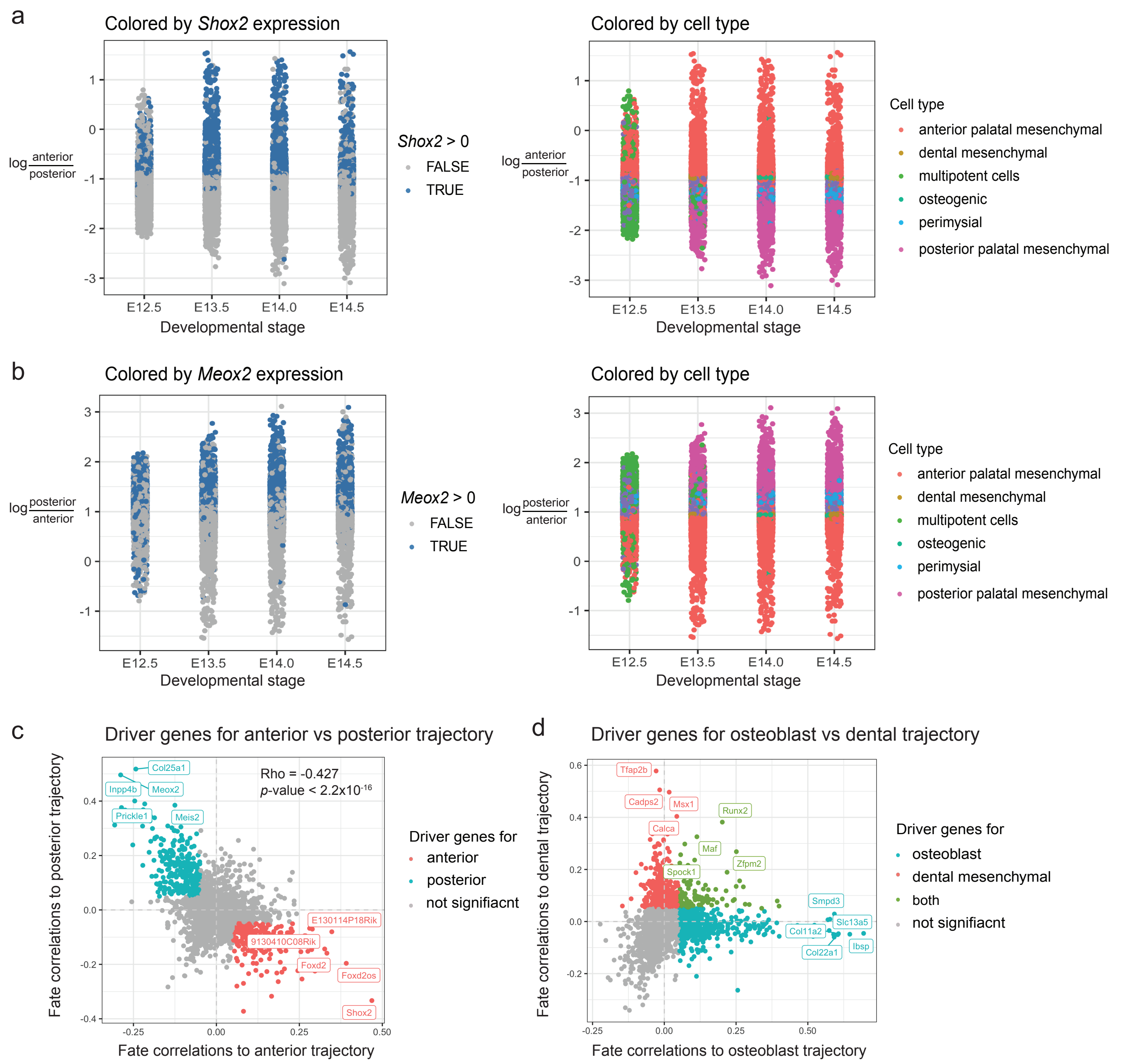

Supplementary Fig. 13. Driver genes for the anterior trajectory show negative fate correlations to the posterior trajectory, which validate the inferences. a Dot plot shows the distribution of log odds of fate probabilities to anterior versus posterior trajectories (y-axis) in each cell across the developmental stage (x-axis). Each dot represents one cell and is colored by *Shox2* expression (left) or cell types (right). Cells with positive expression of *Shox2* also exhibited high fate probabilities to anterior compared to the posterior trajectory. **b** Similar visualization to panel A for log odds of fate probabilities to posterior versus anterior trajectories. Each dot represents one cell and is colored by *Meox2* expression (left) or cell types (right). Cells with positive expression of *Meox2* also exhibited high fate probabilities to anterior compared to the posterior trajectory. **c** Scatter plot shows fate correlations to anterior (x-axis) and posterior (y-axis) trajectories of each gene. Defined driver genes for each trajectory are highlighted in the plot. **d** Scatter plot shows fate correlations to osteoblast (x-axis) and dental mesenchymal (y-axis) trajectories of each gene. Genes in green have similar fate correlations to both trajectories.

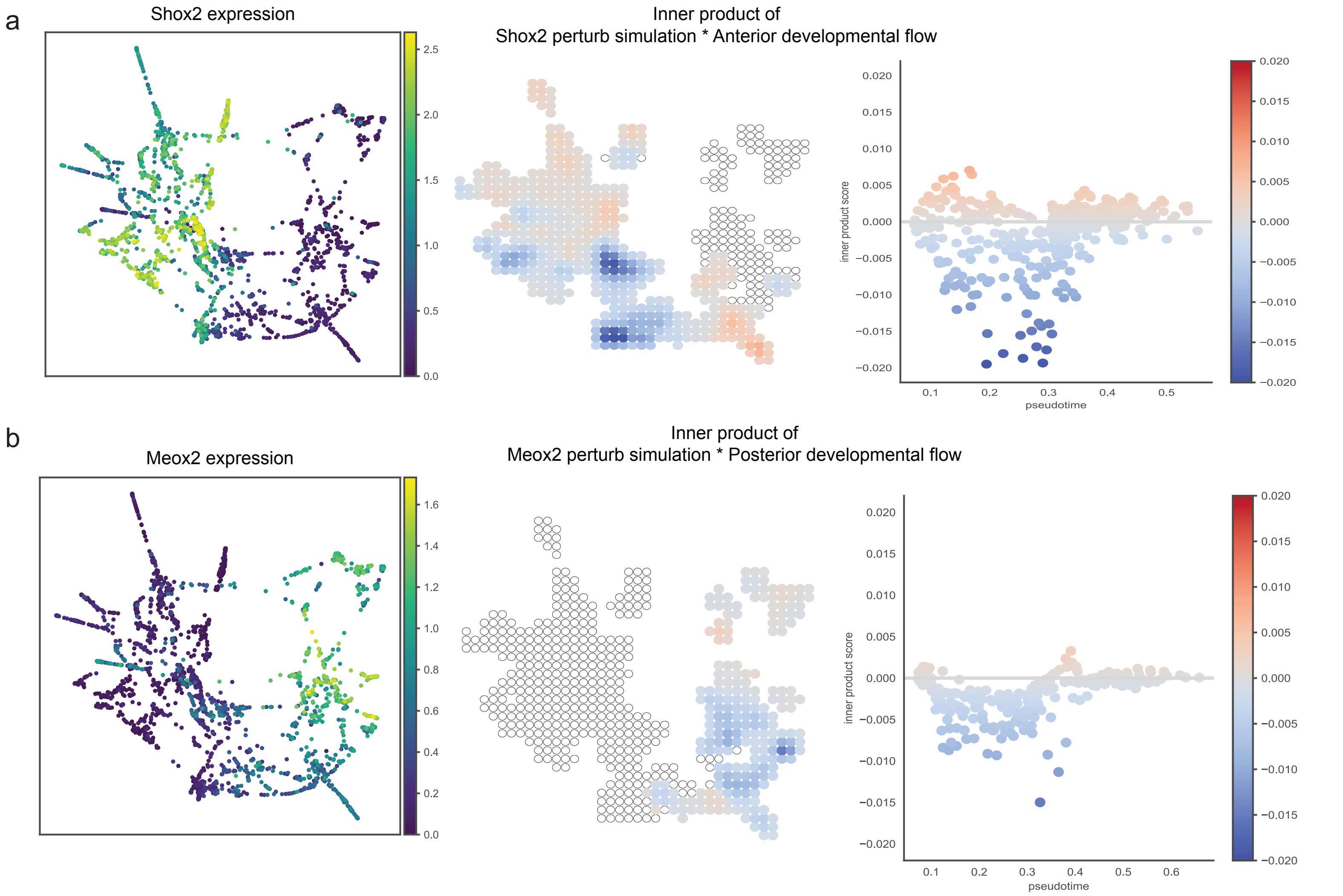

Supplementary Fig. 14. Shox2 and Meox2 exhibit high perturbation score after in silico knockout simulation for anterior and posterior trajectories, respectively. a Left: UMAP visualization of data subset showing Shox2 expression in anterior subpopulation. Middle: Grid plot showing inner product of Shox2 perturb simulation and anterior trajectory developmental flow. Right: scatter plot shows inner product score (y-axis) along pseudotime (x-axis). **b** Same Visualization as panel A for Meox2 in posterior trajectory.

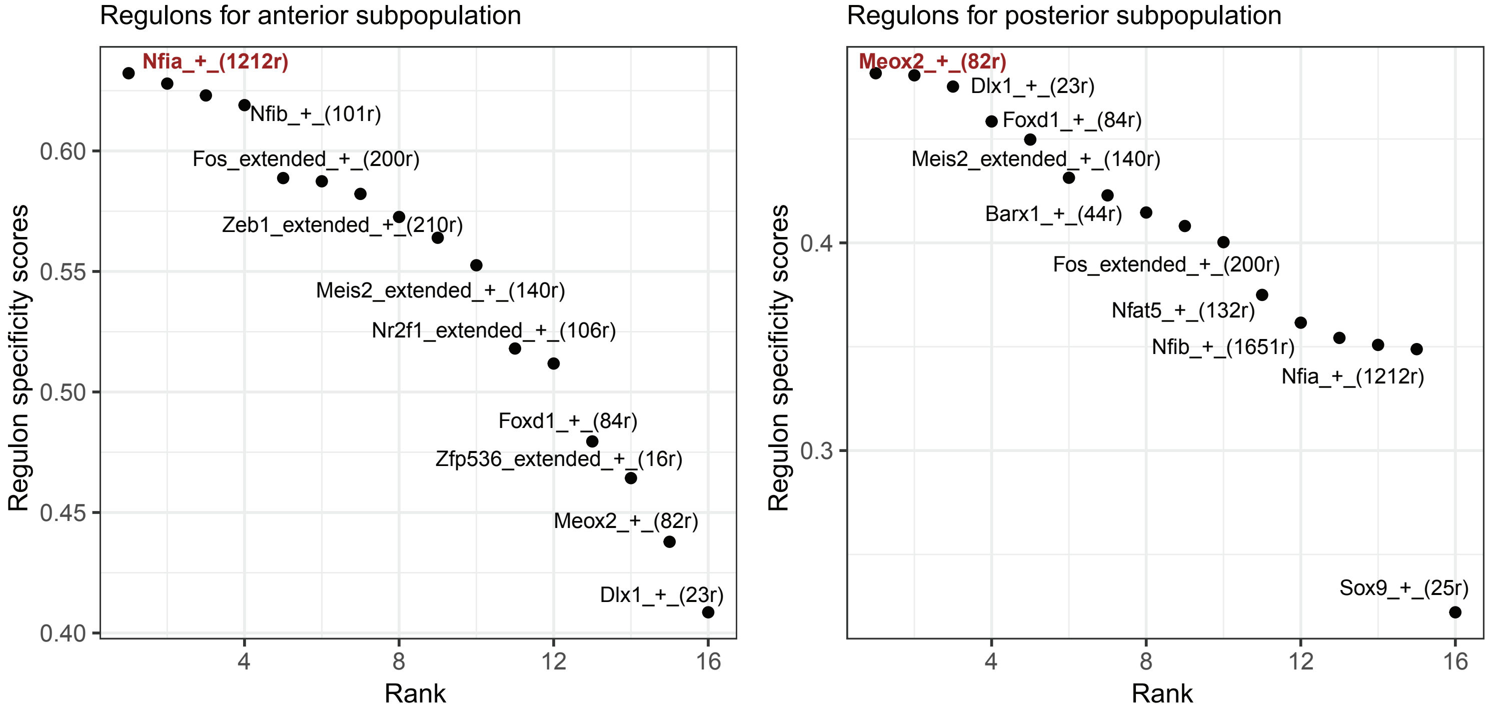

Supplemental Fig. 15. SCENIC+ identified regulons for anterior and posterior subpopulations, respectively. Scatter plot shows regulon specificity score (RSS, y-axis) of ranked regulons (x-axis) in anterior (left) and posterior subpopulations (right).

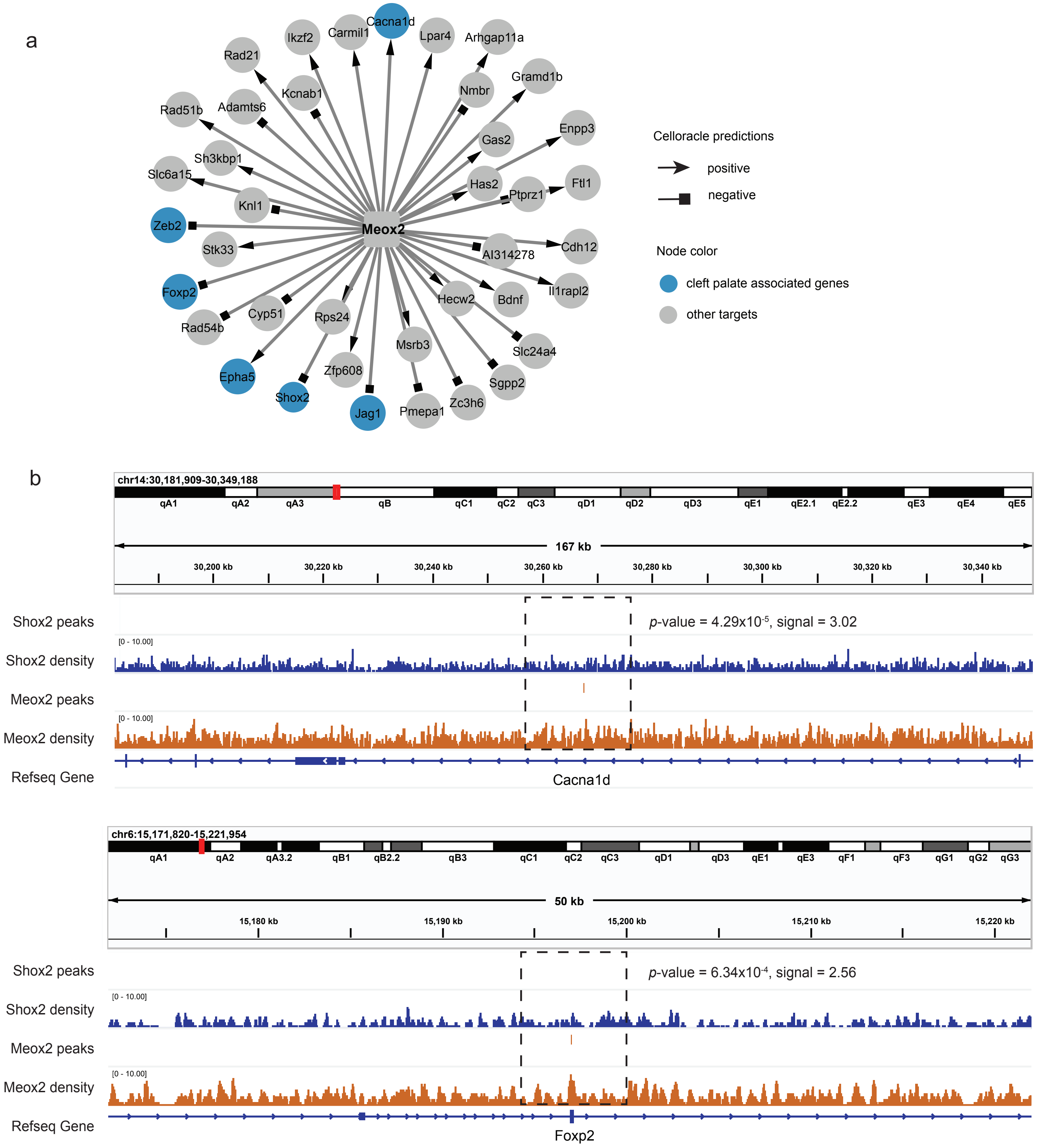

Supplemental Fig. 16. Enlarged Meox2 network identifies target genes associated with cleft palate. a Network graphs visualized using Cytoscape show enlarged predicted MEOX2 regulatory networks (p-value < 0.01). The shape of the arrow heads and target node color is based on the predicted direction of regulation. **b** Integrative Genomics Viewer (IGV) view depicts ChIP-seq binding peak for MEOX2 in the predicted MEOX2 target Cacna1d and Foxp2.

Supplemental Table 1. Quality control metrics for ATAC and GEX libraries in each sample.

Library	ATAC Per Library Metrics			GEX Per Library Metrics			# of cells before preprocessing	# of cells after preprocessing
	Total # of read pairs	# of read pairs per cell	% of reads kept	Total # of read pairs	# of read pairs per cell	% of reads kept		
E12_5_1	253,080,554	37,571	52.90%	175,630,078	26,073	90.00%	6,736	6,348
E12_5_2	201,627,408	34,267	61.40%	144,278,831	24,520	100.00%	5,884	5,498
E13_5_1	189,257,170	86,616	80.60%	176,962,262	80,989	41.50%	2,185	1,693
E13_5_2	217,328,102	89,141	41.00%	156,982,892	64,390	46.60%	2,438	2,226
E13_5_3	209,954,979	43,397	55.40%	174,273,262	36,021	70.50%	4,838	4,527
E14_0_1	194,201,004	40,602	100.00%	156,955,815	32,815	96.30%	4,783	4,496
E14_0_2	232,945,189	53,342	72.50%	168,557,694	38,598	69.40%	4,367	4,112
E14_5_1	173,222,784	37,388	90.70%	192,325,131	41,512	57.80%	4,633	3,889
E14_5_4	206,644,325	54,308	45.50%	153,505,067	40,342	65.70%	3,805	3,365

#: number, %: percent

Supplemental Table 2. PCR primers for quantitative RT-PCR experiments.

Gene	Forward primer	Reverse primer
Cyp26b1	AATTCCATTGGCGACATCCACC	GGTAGCTCTCAAGTGCCTCATG
Efnb2	CCAACAAGACGTCCAGAGCTAG	CCACTTCGGAACCCAGGAGATT
Inhba	GTAAAGTGGGGGAGAACGGG	TTAAGCCCATTTCCTCGGCC
Meox2	GTGGCAGCAAAAGGAAAAGCGAC	GGCAAATTCTGCCTCTAGTTCTC
Nrp1	CGGAGGAATGTTCTGTCGCTATG	GGATAGAACGCCTGAAGAGGAG
Prickle1	AACAGCTCCTGTACCAGTTGCC	CTTCCTCTGAGCACTGAACACC
Satb2	GGGCTAGTGTGTCTCAAGCTG	GAAGTTCTGCATGGCCCTCAG
Shox2	CTATCCAGACGCTTTCATGCGC	ACTGGCTAGCGGCTCCTATAAG
Sim2	CGGAGATCAAGCTCCACAGCAA	CGATCAGGTCTTGTGGCTCATAG
Trps1	CAACCGTTCTGTGCTTTCTGGC	GTGTTGCCTTGGCAATCTGGAG
Gapdh	AACTTTGGCATTGTGGAAGG	ACACATTGGGGGTAGGAACA
