# Supplementary material for: Single-cell multiomics decodes regulatory programs for mouse secondary palate development
Source: Nat Commun. 2024 Jan 27;15:821. doi: 10.1038/s41467-024-45199-x (PMC10821874; doi:10.1038/s41467-024-45199-x)
Supplement: Supplementary file 3 — Description of Additional Supplementary Files [file 41467_2024_45199_MOESM3_ESM.pdf]

Supplementary Data 1 Gene regulatory networks predicted by Celloracle for anterior and posterior trajectories. The get_links function in the Celloracle package is employed for predicting the gene regulatory network, utilizing the "bagging_ridge" modeling method with a bagging number set to 20. The excel table lists each pair of predicted TF and target gene and their coefficient and p-values.
