# Supplementary material for: Single-cell multiomics decodes regulatory programs for mouse secondary palate development
Source: Nat Commun. 2024 Jan 27;15:821. doi: 10.1038/s41467-024-45199-x (PMC10821874; doi:10.1038/s41467-024-45199-x)
Supplement: Supplementary file 5 — Reporting Summary [file 41467_2024_45199_MOESM5_ESM.pdf]

nature portfolio

Corresponding author(s): Dr. Zhongming Zhao

Last updated by author(s): Dec 19, 2023

Reporting Summary

Nature Portfolio wishes to improve the reproducibility of the work that we publish. This form provides structure for consistency and transparency in reporting. For further information on Nature Portfolio policies, see our Editorial Policies and the Editorial Policy Checklist.

Statistics

For all statistical analyses, confirm that the following items are present in the figure legend, table legend, main text, or Methods section.

n/a	Confirmed	
☐	☒	The exact sample size (*n*) for each experimental group/condition, given as a discrete number and unit of measurement
☒	☐	A statement on whether measurements were taken from distinct samples or whether the same sample was measured repeatedly
☐	☒	The statistical test(s) used AND whether they are one- or two-sided<br>*Only common tests should be described solely by name; describe more complex techniques in the Methods section.*
☐	☒	A description of all covariates tested
☐	☒	A description of any assumptions or corrections, such as tests of normality and adjustment for multiple comparisons
☐	☒	A full description of the statistical parameters including central tendency (e.g. means) or other basic estimates (e.g. regression coefficient) AND variation (e.g. standard deviation) or associated estimates of uncertainty (e.g. confidence intervals)
☐	☒	For null hypothesis testing, the test statistic (e.g. *F*, *t*, *r*) with confidence intervals, effect sizes, degrees of freedom and *P* value noted<br>*Give P values as exact values whenever suitable.*
☒	☐	For Bayesian analysis, information on the choice of priors and Markov chain Monte Carlo settings
☒	☐	For hierarchical and complex designs, identification of the appropriate level for tests and full reporting of outcomes
☐	☒	Estimates of effect sizes (e.g. Cohen's *d*, Pearson's *r*), indicating how they were calculated

Our web collection on statistics for biologists contains articles on many of the points above.

Software and code

Policy information about availability of computer code

Data collection	No software was used for data collection.
Data analysis	Custom R (version 3.6.3) and python (version 3) scripts are developed and made available at GitHub. All R and Python scripts supporting the findings of this paper are available on github (https://github.com/fangfang0906/Single_cell_multiome_palate).

For manuscripts utilizing custom algorithms or software that are central to the research but not yet described in published literature, software must be made available to editors and reviewers. We strongly encourage code deposition in a community repository (e.g. GitHub). See the Nature Portfolio guidelines for submitting code & software for further information.

Data

Policy information about availability of data

All manuscripts must include a data availability statement. This statement should provide the following information, where applicable:

- Accession codes, unique identifiers, or web links for publicly available datasets
- A description of any restrictions on data availability
- For clinical datasets or third party data, please ensure that the statement adheres to our policy

The raw and processed single cell multiome data generated in this study have been deposited in the Gene Expression Omnibus database (GEO) under accession code GSE218576 [https://www.ncbi.nlm.nih.gov/geo/query/acc.cgi?acc= GSE218576]. The ChIP-seq data generated in this study are deposited in the GEO under accession code GSE250247 [https://www.ncbi.nlm.nih.gov/geo/query/acc.cgi?acc=GSE250247]. The single cell RNA-seq data from soft palate is available at the GEO

under accession code GSE155928 [https://www.ncbi.nlm.nih.gov/geo/query/acc.cgi?acc=GSE155928]. The raw H3K27 acetylation ChIP-seq data is available at the Sequence Read Archive (SRA) under accession code SRX6976329 [https://www.ncbi.nlm.nih.gov/sra/SRX6976329[accn]].

Research involving human participants, their data, or biological material

Policy information about studies with human participants or human data. See also policy information about sex, gender (identity/presentation), and sexual orientation and race, ethnicity and racism.

Reporting on sex and gender	N/A
Reporting on race, ethnicity, or other socially relevant groupings	N/A
Population characteristics	N/A
Recruitment	N/A
Ethics oversight	N/A

Note that full information on the approval of the study protocol must also be provided in the manuscript.

Field-specific reporting

Please select the one below that is the best fit for your research. If you are not sure, read the appropriate sections before making your selection.

☒ Life sciences ☐ Behavioural & social sciences ☐ Ecological, evolutionary & environmental sciences

For a reference copy of the document with all sections, see nature.com/documents/nr-reporting-summary-flat.pdf

Life sciences study design

All studies must disclose on these points even when the disclosure is negative.

Sample size	Our data was generated at single cell resolution using developing mouse secondary palate at E12.5 (n=2), E13.5 (n=3), E14.0 (n=2), and E14.5 (n=2), which included a total of 37,329 independent cells.
Data exclusions	Cells that don't pass thresholds based on RNA-assay metrics (200< nCount_RNA < 100,000, nFeature_RNA < 7,500, percent.mt < 20) and ATAC-assay metrics (200 < nCount_ATAC < 100,000, nucleosome_signal < 2, TSS.enrichment > 1) were filtered out.
Replication	We profiled 36,154 independent cells from biological replicates. All quality control metrics are reported at each step of the sequencing studies. RNAscope in situ hybridization was conducted in six independent experiments, each yielding consistent results.
Randomization	Samples at the same developmental stage were allocated to same group.
Blinding	The dimension reduction is unsupervised and blinding was not relevant to our study.

Reporting for specific materials, systems and methods

We require information from authors about some types of materials, experimental systems and methods used in many studies. Here, indicate whether each material, system or method listed is relevant to your study. If you are not sure if a list item applies to your research, read the appropriate section before selecting a response.

Materials & experimental systems

n/a	Involved in the study	
☐	☒	Antibodies
☒	☐	Eukaryotic cell lines
☒	☐	Palaeontology and archaeology
☐	☒	Animals and other organisms
☒	☐	Clinical data
☒	☐	Dual use research of concern
☒	☐	Plants

Methods

n/a	Involved in the study	
☐	☒	ChIP-seq
☒	☐	Flow cytometry
☒	☐	MRI-based neuroimaging

Antibodies

Antibodies used	see Methods section in manuscript, all antibodies are listed in detail under the method.
Validation	specificity of these antibodies has been tested by the manufacturers.

Animals and other research organisms

Policy information about studies involving animals; ARRIVE guidelines recommended for reporting animal research, and Sex and Gender in Research

Laboratory animals	C57BL/6J mice (000664, Jackson Laboratory) at embryonic day (E)12.5, E13.5, E14.0, and E14.5
Wild animals	No wild animals were used in the study.
Reporting on sex	As we are using mouse embryos for experiments, sex can't be determined.
Field-collected samples	No field-collected samples were used in the study.
Ethics oversight	The protocol was approved by the Animal Welfare Committee (AWC) and the Institutional Animal Care and Use Committee (IACUC) of UTHealth (AWC 22-0087).

Note that full information on the approval of the study protocol must also be provided in the manuscript.

Plants

Seed stocks	N/A
Novel plant genotypes	N/A
Authentication	N/A

ChIP-seq

Data deposition

☒ Confirm that both raw and final processed data have been deposited in a public database such as GEO.

☒ Confirm that you have deposited or provided access to graph files (e.g. BED files) for the called peaks.

Data access links<br>*May remain private before publication.*	The raw and processed data were deposited at the Gene Expression Omnibus database (GEO) under accession code GSE218576. The secure token has been created to allow review of record GSE218576 while it remains in private status. Go to https://nam04.safelinks.protection.outlook.com/?url=https%3A%2F%2Fwww.ncbi.nlm.nih.gov%2Fgeo%2Fquery%2Facc.cgi%3Facc%3DGSE218576&data=05%7C01%7CFangfang.Yan.1%40uth.tmc.edu%7C20d627fcf4154e71a32408db15682d89%7C7b326d2441ad4f57bc6089e4a6ac721b%7C0%7C0%7C638127309409664865%7CUnknown%7CTWFpbGZsb3d8eyJWIjoiMC4wLjAwMDAiLCJQIjoiV2luMzIiLCJBTiI6Ik1haWwiLCJXVCI6Mn0%3D%7C3000%7C%7C%7C&sdata=wjgMcpXjR1ENHS4SxwxZbfmgTjJurdGUZoEAA9SvwAA%3D&reserved=0<br>Enter secure token wxkfmmssbvqldkf into the box.
Files in database submission	N/A
Genome browser session<br>(e.g. UCSC)	N/A

Methodology

Replicates	Two independent ChIPs were conducted for library generation for each group.
Sequencing depth	paired-end
Antibodies	SHOX2 and MEOX2 antibodies
Peak calling parameters	macs2 callpeak -t Shox2_rep1.sorted.bam Shox2_rep2.sorted.bam -f BAM -B -g mm -n Shox2_merged -p 0.01
Data quality	Only peaks passed p-value threshold of 0.01 were called.
Software	The raw FASTQ datasets were preprocessed using FastQC (version 0.12.1) and mapped to the mouse genome (mm10) using the Bowtie2 alignment algorithm (version 2.5.1). The resulting alignment files were then used as input for MACS2 (version 2.2.9.1) to call

peaks with a p-value threshold of 0.01. Subsequently, the generated bed files were then converted to bigwig files and visualized using Integrative Genomics Viewer (IGV).
